# Antithymocyte globulin is associated with a lower incidence of de novo donor‐specific antibody detection in lung transplant recipients: A single‐center experience

**DOI:** 10.1002/iid3.491

**Published:** 2021-07-26

**Authors:** Tathagat Narula, Samir Khouzam, Francisco Alvarez, David Erasmus, Zhuo Li, Yousif Abdelmoneim, Mohamed Elrefaei

**Affiliations:** ^1^ Department of Transplantation Mayo Clinic Jacksonville Florida USA; ^2^ Department of Laboratory Medicine and Pathology Mayo Clinic Jacksonville Florida USA; ^3^ Health Sciences Research Mayo Clinic Jacksonville Florida USA

## Abstract

**Purpose:**

Induction immunosuppression has improved the long‐term outcomes after lung transplant. This is the first report exploring the association of induction immunosuppression with the development of de novo donor‐specific human leukocyte antigen (HLA) antibodies (DSA) in lung transplant recipients (LTR).

**Methods:**

Sixty‐seven consecutive primary LTR were followed for 3 years posttransplant. A total of 41/67 (61%) LTR‐received induction immunosuppression using a single dose of rabbit Antithymocyte Globulin (rATG; 1.5 mg/kg) within 24 h of transplant. All recipients had a negative flow cytometry crossmatch on the day of transplant. Serum samples at 1, 3, 6, and 12 months posttransplant were assessed for the presence of de novo HLA DSA.

**Results:**

De novo HLA DSA were detected in 22/67 (32.8%) LTR within 1‐year posttransplant. Of these, 9/41 (21.9%) occurred in the induction therapy group and 13/26 (50%) in the noninduction group. Class II DSA were detected in 3/41 (7.3%) LTR who received induction compared to 9/26 (34.6%) LTR without induction immunosuppression (*p* = .005). Differences in overall survival or freedom from chronic lung allograft dysfunction rates between the two groups were not statistically significant.

**Conclusion:**

Induction immunosuppression utilizing a modified regimen of single‐dose rATG is associated with a significant reduction in de novo DSA production in LTR.

## Graphical abstract

Induction immunosuppression utilizing a modified single‐dose antithymocyte globulin is associated with a significant reduction in de novo DSA production post lung transplant

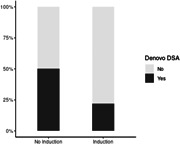

## 1 INTRODUCTION

While there has been an overall increase in lung transplants, long‐term survival after lung transplant remains abysmal, with a 5‐year survival rate of approximately 55%.^1^ Poor long‐term survival is primarily driven by the onset of chronic lung allograft dysfunction (CLAD). Manifesting as two different phenotypes, CLAD can present as an inflammatory process in noncartilaginous airways‐bronchiolitis obliterans (BOS) or, less commonly, as a restrictive allograft syndrome (RAS) with peripheral lung fibrosis.^2^

Among the many proposed risk factors, de novo donor‐specific antibodies (DSA) human leukocyte antigen (HLA) are associated with BOS development in lung transplant recipients (LTR).^3^, ^4^ HLA antigens are divided based on their structure and function into HLA Class I and Class II. As seen in other solid organ transplantation, de novo DSA can cause antibody‐mediated rejection (AMR), resulting in increased graft loss and decreased patient survival.^5^, ^6^, ^7^ While the precise causal relationship between DSA and these morbidities is unclear, it is evident that DSA formation precedes graft dysfunction. LTR who clear DSA following immunomodulatory therapy have similar AMR and BOS incidence as those who do not develop DSA. Besides, recipients who clear DSA have a lower incidence of BOS and better survival than those with persistent DSA.^8^

Current strategies for AMR treatment rely on using a combination of nonstandardized immunomodulatory therapies to address multiple pathophysiologic pathways. Ideally, management strategies should incorporate preventative measures to reduce the risk of DSA development. On this front, induction immunosuppression, entailing the administration of an intense and potent immunosuppressive agent such as the polyclonal antibody preparation (equine or rabbit anti‐thymocyte globulin [ATG]) in the perioperative or immediate postoperative period may have a role in mitigating the risk of development of DSA. The last decade has witnessed a steady increase in induction therapy utilization with lung transplantation. In the most recent iteration of the International Society of Heart and Lung Transplantation (ISHLT) adult lung transplant registry, 80% of patients who received a lung transplant between 1/2018 and 6/2018 received induction therapy, a significant increase compared to the 50% utilization of induction immunosuppression in LTR in 2010.^9^ At our institution, the practice during the study period of January 2016 to December 2017 entailed induction immunosuppression after lung transplant with single‐dose rATG. We withheld induction when the risk of infection or noninfectious complications from induction outweighed the benefits.

rATG is a purified and pasteurized immunoglobulin G (IgG) obtained from rabbits immunized with human thymocytes. It depletes circulating T‐cells, modulates T‐cell activation and cytotoxic activities.^10^ The traditional rationale behind induction is to delay initiation of maintenance immunosuppression, reduce its cumulative dose, and decrease acute cellular rejection (ACR).^11^, ^12^ Induction immunosuppression can decrease the incidence of acute rejection, reduce or delay BOS onset and improve graft and patient survival.^13^, ^14^, ^15^, ^16^ Induction immunosuppression using rATG in deceased donor kidney transplant recipients is associated with a lower incidence of de novo DSA, AMR, and increased graft survival.^17^, ^18^, ^19^, ^20^ However, the impact of induction with rATG on de novo DSA after lung transplant remains unexplored. We hypothesized that induction immunosuppression would decrease de novo HLA DSAs and improve lung transplant outcomes. This study aimed to assess the impact of induction immunosuppression using a single dose of rATG on de novo HLA DSA, BOS, and patient survival postlung transplantation.

## 2 MATERIAL AND METHODS

### 2.1 Study subjects and source of samples

**Figure 1:**
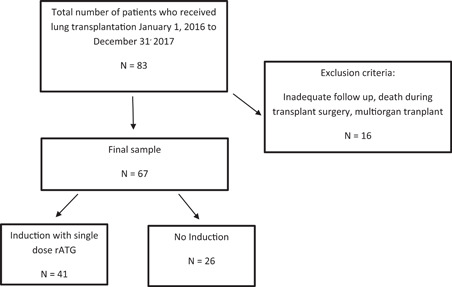
Flow chart showing lung transplant recipients included in the study

All patients who underwent lung transplant at Mayo Clinic in Florida between January 2016 and December 2017 were assessed pre‐ and post‐transplant for the presence of HLA DSA per our institutional protocol. Inclusion criteria included all primary and repeat LTR that were greater than 18 years old irrespective of gender and race. Recipients who did not survive the intra‐operative period during lung transplant surgery or recipients undergoing multiorgan transplantation were excluded from the study (Figure 1). Demographic and clinical information (age, gender, ethnicity, smoking history, patient underlying lung disease diagnosis, comorbidities), transplant data (date and type of transplant, number of transplants, donor ID, and lung allocation score), physiologic parameters (PFT, 6 min walk), pathology posttransplant, immunosuppression therapy, HLA lab test results (HLA antibodies and crossmatch results), and date of death whenever applicable was obtained from the medical records. The study was approved by the Institutional Review Board/Human Subjects Division at Mayo Clinic, and all study participants gave written informed consent for the anonymous use of test results in research studies. All LTR had a negative cytotoxicity, and flow cytometry crossmatches on the day of transplant. All LTR were tested for the presence of de novo HLA class I and II DSA at 1, 3, 6, and 12 months posttransplant.

CLAD was defined, per ISHLT diagnostic criteria, as a persistent decline of more than 20% in the measured forced expiratory volume in 1 second (FEV1) value from the baseline value. The baseline value was computed as the mean of the two best postoperative FEV1 measurements taken at least 3 weeks apart. CLAD can present either as a predominantly obstructive ventilatory pattern (BOS), a restrictive pattern (RAS), or a mixed obstructive and restrictive pattern. Restriction was defined as a ≥10% reduction in baseline TLC combined with parenchymal opacities and/or increasing pleural thickening likely to cause a restrictive physiology.^21^ All patients in the study were treated posttransplant with a standard immunosuppressive regimen incorporating systemic corticosteroids, calcineurin inhibitor, and an antimetabolite.

### 2.2 HLA antibody detection

Recipient serum samples were treated with ethylenediaminetetraacetic acid to avoid the prozone effect and tested for IgG antibodies against HLA class I and II using the LABScreen single antigen beads (One Lambda‐ThermoFisher, Inc.) per manufacturer protocol. Briefly, microbeads are coated with 100 purified HLA class I or II antigens and preoptimized reagents to detect HLA antibodies in human sera. A negative control serum is used to establish the background value for each bead in a test batch. Test serum is incubated with LABScreen beads. Any HLA antibodies present in the test serum bind to the antigens on the beads and then are labeled with R‐Phycoerythrin (PE)‐conjugated goat anti‐human IgG. The LABScan flow analyzer simultaneously detects the fluorescent emission of PE and a dye signature from each bead. The presence of DSA against HLA‐A, ‐B, ‐C, ‐DRβ, ‐DQα/β, and ‐DPβ antigens is determined. DSA were defined as having a Mean Fluorescence Intensity of more than 1000. A persistent HLA DSA were defined as having two or more positive antibody tests for the same HLA antigen at least 1 month apart

### 2.3 HLA genotyping

DNA samples were extracted from peripheral blood mononuclear cell or lymph node cell samples by the salting method (Qiagen) according to the manufacturer's instructions.^22^ According to the manufacturer's instructions, HLA genotyping was performed by reverse polymerase chain reaction sequence specific oligonucleotide probe (One Lambda).

### 2.4 Statistical analysis

Continuous variables were summarized as mean (*SD*) and median (range), while categorical variables were reported as frequency (percentage). Continuous variables were compared between patients with and without induction using the Wilcoxon rank‐sum test, and categorical variables were compared with the *χ* ^2^ test. Kaplan‐Meier method was used to estimate 1, 2, and 3‐year survival, freedom from BOS, and BOS‐free survival rates. Cox regression models were used to evaluate the impact of induction on long‐term outcomes after adjusting for age and LAS. All tests were two‐sided, with an *α* level set at 0.05 for statistical significance.

## 3 RESULTS

### 3.1 Patient demographics and clinical characteristics

Sixty seven consecutive LTR between January 2016 and December 2017 were included in the study Baseline demographic and clinical characteristics of the study participants are summarized in Table 1. A total of 41/67 (61%) LTR received Induction immunosuppression using a single dose rATG (1.5 mg/kg) within 24 h of transplant. The median age of LTR that received Induction immunosuppression was significantly higher compared to LTR with no induction (65 vs. 57 respectively; *p* = .031). The median lung allocation score (LAS) was significantly higher in LTR that did not receive Induction immunosuppression (58.4 vs. 40.1; *p* = .003). In the noninduction group, a significantly higher fraction of patients received bilateral lung transplants (73.1% vs. 48.8%; *p* = .049). There were no significant differences in the number of HLA antigen mismatches and the number of LTR with DSA at the time of transplant between the two groups.

**Table 1:** Demographics and clinical characteristics of study participants^a^

	No induction (*N* = 26)	Induction (*N* = 41)	Total (*N* = 67)	*p* value
Age^b^	57 (21–76)	65 (34–76)	62 (21–76)	.031
Gender (M/F ratio)	1.36:1	3.1:1	2.19–1	.123
BMI^c^	24 (18–32)	26 (16–36)	25 (16–36)	.412
DSA^d^	9 (34.6%)	17 (41.5%)	26 (38.8%)	.575
LAS^e^	58.4 (35.3–92.8)	40.1 (32.5–90.3)	45.1 (32.5–92.8)	.003
HLA matches^f^	3.3 (1.8)	3.1 (2.1)	3.2 (2.0)	.625
CPRA^g^	22.5 (31.5)	18.7 (27.6)	20.2 (29.0)	.796
Lung disease categories^h^				
(A) Obstructive	3 (11.5%)	11 (26.8%)	14 (20.9%)	
(B) Vascular	0	0	0	
(C) Infectious	3 (11.5%)	1 (2.4%)	4 (6%)	
(D) Restrictive	20 (76.9%)	29 (70.7%)	49 (73.1%)	
Specific diagnosis^i^				
Bronchiectasis^1^	1 (3.8%)	0 (0.0%)	1 (1.5%)	
CF^2^	2 (7.7%)	1 (2.4%)	3 (4.5%)	
PF, other^3^	3 (11.5%)	3 (7.3%)	6 (9.0%)	
CLAD‐retransplant^4^	2 (7.7%)	2 (4.8%)	4 (6%)	
COPD^5^	0 (0.0%)	8 (19.5%)	8 (11.9%)	
Constrictive bronchiolitis^6^	0 (0.0%)	1 (2.4%)	1 (1.5%)	
Sarcoidosis^7^	3 (11.5%)	1 (2.4%)	4 (6.0%)	
Obliterative bronchiolitis^8^	1 (3.8%)	0 (0.0%)	1 (1.5%)	
CTD–ILD^9^	1 (3.8%)	0 (0.0%)	1 (1.5%)	
IPF^10^	13 (50.0%)	25 (61.0%)	38 (56.7%)	
Laterality^j^				.049
Single	7 (26.9%)	21 (51.2%)	28 (41.8%)	
Bilateral	19 (73.1%)	20 (48.8%)	39 (58.2%)	

a Data shown at the time of transplant.

b Age. median values and range in between brackets.

c Body mass index. median values and range in between brackets.

d Number and percent of lung transplant recipients with donor specific HLA antibodies detected.

e Lung allocation score median values and range in between brackets.

f Mean and *SD* of the number of HLA class I and II matches between lung transplant recipients and donors.

g Mean values and *SD* in between brackets.

h Diagnosis by UNOS listing category. Number and percent of lung transplant recipients with (A) obstructive lung disease (B) pulmonary vascular disease (C) infectious disease (D) restrictive lung disease.

i Specific lung disease diagnosis. Number and percent of lung transplant recipients with ^1^Bronchiectasis, ^2^Cystic fibrosis, ^3^Pulmonary fibrosis—other causes, ^4^Chronic lung allograft dysfunction—lung retransplant, ^5^Chronic obstructive pulmonary disease, ^6^Constrictive bronchiolitis, ^7^Sarcoidosis, ^8^Obliterative bronchiolitis, ^9^Connective tissue disease‐associated interstitial lung disease, and ^10^Idiopathic pulmonary fibrosis.

j Number of recipients received single or double lung transplant and percent in between brackets.

### 3.2 Effect of induction immunosuppression on de novo HLA DSA postlung transplantation

**Figure 2:**
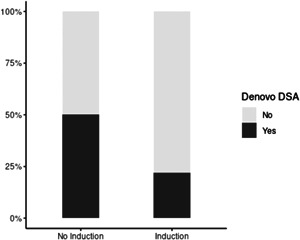
Detection of de novo DSA postlung transplant. Persistent de novo HLA DSA were detected in 9/41 (21.9%) compared to 13/26 (50%) LTR with and without induction immunosuppression, respectively at 12 months posttransplant (*p* = .017). DSA, donor‐specific HLA antibody; LTR, lung transplant recipient

De novo HLA DSA were detected in 22/67 (32.8%) LTR within 1‐year posttransplant (Table 2). They were detected in 9/41 (21.9%) compared to 13/26 (50%) LTR with and without induction immunosuppression, respectively (Figure 2; *p* = .017). HLA class II DSA were detected in 3/41 (7.3%) compared to 9/26 (34.6%) LTR with and without Induction immunosuppression, respectively (*p* = .005). We observed no significant difference in the median mean fluorescence intensity (MFI) levels of de novo HLA Class I and II DSA between the two groups (*p* = .197 and *p* = .466 for Class I and II, respectively). Similarly, the cumulative MFI values of de novo HLA Class I and II DSA were not significantly different between LTR with and without induction immunosuppression (data not shown). The mean number of days for de novo DSA detection was 136 and 178 for LTR with and without Induction immunosuppression, respectively (*p* = .452).

**Table 2:** Detection of de novo DSA post‐lung transplantation

	No induction (*N* = 26)	Induction (*N* = 41)	Total (*N* = 67)	*p* value
Total de novo DSA^a^	13 (50%)	9 (22%)	22 (32.8%)	.017
Class I only	3 (11.5%)	2 (4.9%)	5 (7.5%)	.312
Class II only	9 (34.6%)	3 (7.3%)	12 (17.9%)	.005
Class II or I and II^b^	10 (38.5%)	7 (17.1%)	17 (25.4%)	.05
MFI of class I DSA^c^	1968 (1432–4746)	1379 (1000–2312)	1680 (1000–4746)	.197
MFI of class II DSA^c^	3260 (1244–4927)	1964 (1214–5565)	2743 (1214–5565)	.466
Days to DSA^d^	178 (126)	136 (133)	161 (128)	.452

Abbreviations: DSA, donor‐specific HLA antibody; MFI, mean fluorescence intensity.

^a^ Number and percent of LTR with de novo HLA DSA.

^b^ Total number and percent of LTR with either de novo HLA class II only or both Class I and II DSA.

^c^ Median and range of MFI.

^d^ Mean and *SD* of the number of days post‐transplant for detection of de novo DSA.

### 3.3 Induction immunosuppression, survival, and CLAD

**Figure 3:**
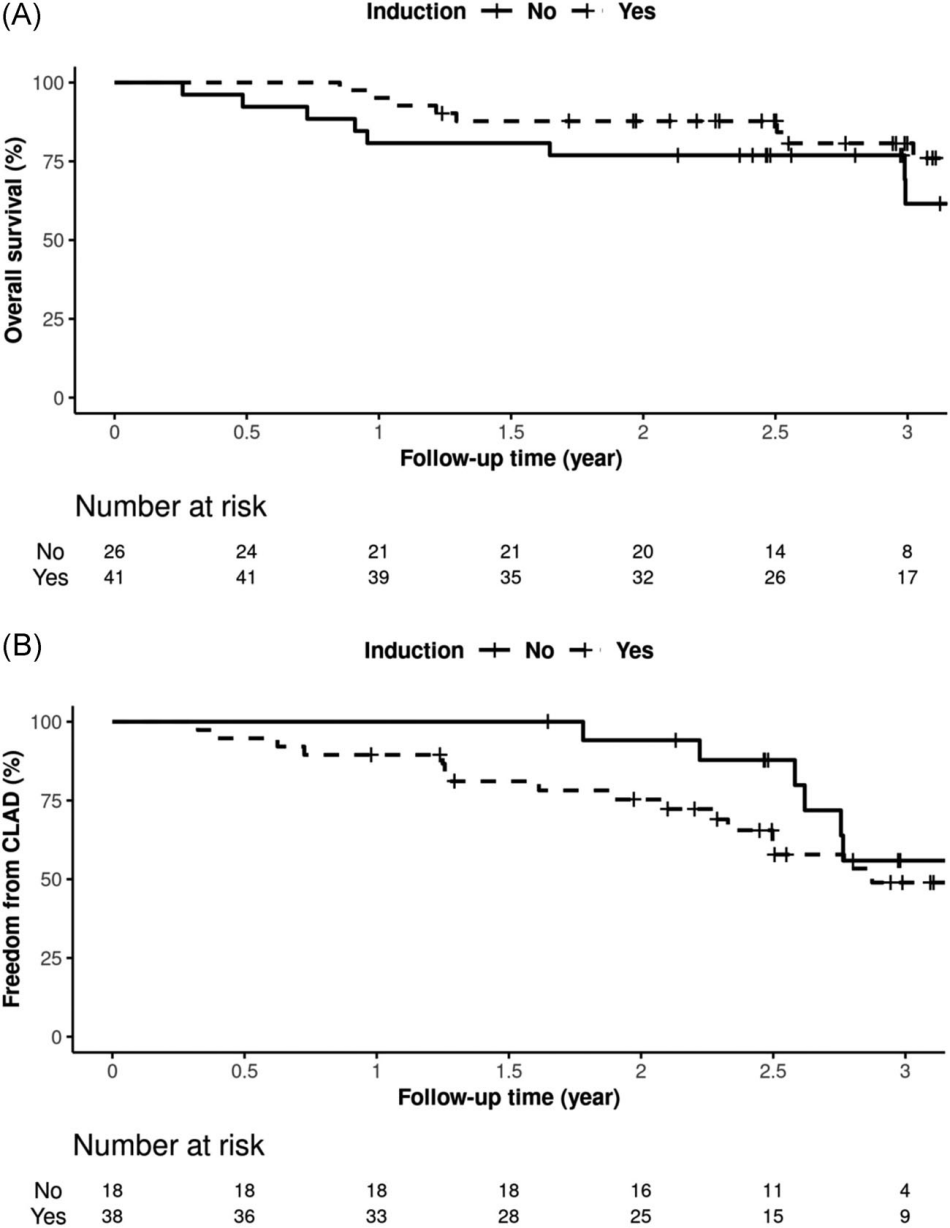
The Kaplan–Meier curve of (A) overall percent survival and (B) percent freedom from CLAD up to 3 years posttransplant. The 3‐year percent survival rates were 80.7% (68.6–95) and 61.5% (42.3–89.5) for LTR with and without induction immunosuppression, respectively. The 3‐year percent freedom from CLAD rates were 49% (34–71) and 56% (35–90) for LTR with and without induction immunosuppression, respectively. Differences were not statistically significant (*p* > .05). CLAD, chronic lung allograft dysfunction; LTR, lung transplant recipient

Among LTR who received induction, 10/41 (24.4%) died within 3 years posttransplant compared to 9/26 (34.6%) without induction immunosuppression. The causes of death are summarized in Table 3. The overall three‐year percent survival rates were 80.7% (95% confidence interval [CI]: 68.6%–95%) and 61.5% (95% CI: 42.3%–89.5%) for LTR with and without induction immunosuppression, respectively (Figure 3A). The presence of CLAD was assessed in 56/67 LTR. CLAD was diagnosed in 22/56 (39.3%) LTR within 3 years posttransplant. CLAD was ungradable in 11/67 patients due to airway stenosis or the presence of a tracheostomy tube. Among patients with CLAD, 18/22 (81.8%) had BOS, 3/22 (13.6%) had RAS, 1/22 (4.5%) had a mixed obstructive and restrictive phenotype. The 3‐year freedom from CLAD rates were 49% (95% CI: 34%–71%) and 56% (95% CI: 35%–90%) for LTR with and without induction immunosuppression, respectively (Figure 3B). The differences in overall survival and freedom from CLAD rates between LTR and without induction immunosuppression were not statistically significant before or after adjusting for age or LAS differences.

**Table 3:** Summary of cause of death

Cause of death	No Induction (*N* = 9)	Induction (*N* = 10)
Acute cellular rejection	0	2
Acute peritonitis	1	0
ARDS	1	1
Bacterial Pneumonia	0	1
CLAD	1	3
CMV pneumonitis	1	0
End‐stage liver disease	1	1
Humoral rejection	1	1
Ischemic colitis	1	0
Massive hemoptysis	1	0
Myelodysplastic syndrome	1	0
Non‐small cell lung cancer	0	1

Abbreviations: ARDS, acute respiratory distress syndrome CLAD, chronic lung allograft dysfunction; CMV, cytomegalovirus.

## 4 DISCUSSION

This is the first report exploring the association of induction immunosuppression with de novo DSA production and long term clinical outcomes. We report a significant reduction in de novo DSA production in LTR who received a modified regimen of a single‐dose rATG induction immunosuppression. A better understanding of antibody‐mediated allograft damage in LTR in the last few years has translated into multiple investigations highlighting the deleterious effects of de novo DSA in this population. Clinically, in the same time frame, we have witnessed an increased use of induction immunosuppression in LTR. Among the various induction agents used, the proportion of LTR receiving interleukin‐2 antagonists has increased over time, whereas polyclonal rATG or alemtuzumab use is less common in recent years.^9^ Concerns about rATG use have stemmed from its adverse effect profile with significant hematologic side effects (cytopenias), increased risk of opportunistic infections. and increased de novo posttransplant malignancy.^23^ Trials using rATG in LTR have typically employed a multidose regimen of 3–4 doses administered at 24‐h intervals.^24^ To mitigate the adverse effect profile of rATG, we switched our induction strategy from a three‐dose regimen to a single dose of ATG (1.5 mg/kg) administered in the first 72 h after transplantation.

We detected de novo HLA DSA in 32.8% of recipients within 1 year postlung transplant, which is consistent with previously published data. The prevalence of de novo DSA varies significantly among different studies ranging from 12% to 47%.^3^, ^4^, ^25^, ^26^ This variability reflects differences in the study population and the methodology used for HLA antibody detection. We observed a significant reduction in total DSA among recipients of induction that was driven by significantly lower DSA in patients with a combination of Class I and Class II and those with Class II DSA only. The mitigation of DSA in these categories is critical because the presence of de novo class II or both Class I + II DSA, especially DQ‐specific DSA, is associated with increased AMR and CLAD.^25^, ^27^, ^28^, ^29^, ^30^ The ability to ameliorate these particular DSA adds further intrigue and promise to the potential benefits of the abbreviated rATG induction regimen in LTR. Mechanistically, the amelioration of antibody responses by rATG may be mediated by complement‐independent apoptosis of memory B cells, plasma cells, and donor‐specific memory T cells.^31^, ^32^ In addition, rATG has been shown to induce regulatory CD4 + T cells that may inhibit DSA production by B cells.^33^, ^34^, ^35^

We did not observe significant differences in the median or cumulative MFI values of de novo HLA DSA between the two study groups. However, these parameters are approximates and may not accurately represent the complex antigen‐antibody interaction in vivo. The deleterious effects of de novo DSA are well established in the literature. However, we did not observe a significant difference in overall survival or freedom from CLAD between the two groups despite the significant reduction in de novo DSA with induction. The relatively small number of LTR in the study and short followup time may have limited our ability to detect significant differences in overall survival or freedom from CLAD. In addition, other variables may have balanced out the difference in long‐term outcomes between the two groups. The cohort that received induction immunosuppression was significantly older while a significantly higher percentage of LTR in the noninduction group received bilateral lung transplant. ISHLT registry data reveals that the risk of 1 year posttransplant mortality increases progressively beyond age 55.^36^ Multiple other studies corroborate that older recipients have worse survival postlung transplant.^37^, ^38^, ^39^, ^40^ In addition, median survival for bilateral lung transplant is significantly higher than single lung transplant (7.8 vs. 4.8 years, respectively).^9^ The lack of significant differences in overall survival and freedom from CLAD rates between the two groups despite a significantly lower percentage of bilateral lung transplants in the induction group suggest potential benefit from a single‐dose rATG induction. We postulate that the negative implications of older age and higher percentage of single lung transplants in the induction group were neutralized out by significantly higher LAS in the noninduction group that is associated with increased morbidity and mortality posttransplant.^41^

The single‐center nature of this study poses obvious limitations. We also recognize that any observed associations are not definitive and do not establish cause and effect. In vitro immunologic data often do not adequately address and mirror the complexity of in vivo immune processes. However, this study provides novel insights on the association of induction immunosuppression using single‐dose rATG with de novo DSA production in a previously unstudied population of LTR. It also raises further queries in the complicated milieu of transplant immune interactions. The spectrum of commonly used induction agents in LTR includes alemtuzumab and basiliximab in addition to rATG. We have incorporated these alternate regimens in our practice in the last three years. We are currently investigating the comparative effects of different induction agents on de novo DSA production in LTR based on the results from this study.

While HLA DSA are deemed the primary mediators of AMR in lung allografts, the presence of antibodies to self‐antigens (SAgs), such as collagen‐V (Col‐V) and K‐α−1‐tubulin (KA1T), has been reported to negatively impact lung allograft outcome by increasing the risk of primary graft dysfunction and BOS.^42^, ^43^, ^44^, ^45^, ^46^, ^47^, ^48^, ^49^, ^50^ A synergistic negative impact of HLA DSA and antibodies against SAgs on allograft function development of BOS has also been proposed.^51^ These observations merit longitudinal studies exploring effect of induction immunosuppression and antibodies against SAgs.

## 5 CONCLUSION

Induction immunosuppression utilizing a modified single‐dose rATG is associated with a significant reduction in de novo DSA production in LTR. Comparable long‐term outcomes despite a higher proportion of single LTR in an older cohort suggest clinical benefit from induction immunosuppression. Our results create the foundation for future studies to explore the association of various induction immunosuppression regimens with the development of de novo DSA and antibodies against SAgs in LTR.

## AUTHORS CONTRIBUTIONS

Tathagat Narula, Samir Khouzam, Francisco Alvarez, David Erasmus, Yousif Abdelmoneim, and Mohamed Elrefaei acquired, analyzed, and interpreted the patient data. Zhuo Li performed statistical analysis. Tathagat Narula and Mohamed Elrefaei were major contributors in writing the manuscript. All authors read and approved the final manuscript.

## CONFLICT OF INTERESTS

The authors declare that there are no conflict of interests.

## ETHICAL APPROVAL

The study was approved by the Institutional Review Board/Human Subjects Division at Mayo Clinic, and all study participants gave written informed consent for the anonymous use of test results in research studies.

## Data Availability

The data sets used and/or analyzed during the current study are available from the corresponding author on reasonable request.
